# Upper-air meteorological dataset for Uyo, using radiosonde

**DOI:** 10.1016/j.dib.2023.108904

**Published:** 2023-01-13

**Authors:** Opeyemi Adamson Oloyede, Nicolas Lopez, Simeon Ozuomba, Philip Asuquo, Emmanuel Essien, Alexander Agbu

**Affiliations:** a Advanced Space Technology Applications Laboratory Uyo, National Space Research and Development Agency, Federal Capital Territory, Abuja, Nigeria; b Department of Electrical/Electronic and Computer Engineering, University of Uyo, Akwa Ibom, Uyo, Nigeria; c Kanda Weather Group LLC, 11009N Lewis Avenue, Kansas City, MO 64157, USA; d Department of Data Science, University of Salford, Greater Manchester, Salford, United Kingdom

**Keywords:** Climate change, Environmental risk assessment, Weather balloon, Weather Prediction, GIS

## Abstract

Weather pattern anomalies and climate change have greatly impacted human activities and the environment in varying ways. Whether induced naturally or by anthropogenic activities, it remains a menace to global public health. A foreknowledge of the weather/climate change can help in mitigating the impact of disasters emanating from these changes. Upper-air meteorological data play an exceptionally large role in weather and climate prediction. However, there is a paucity of ground truth meteorological data in Nigeria and many parts of Africa. Consequently, the need to measure and archive these data. Internet of things and blockchain technologies are employed to build a system that captures and records meteorological data at up to 9,000 metres above sea level. Spanning between January 18, 2021 and July 26, 2021, in Uyo local government area, upper air pressure, temperature, dew point, time and the elevation at which they were captured, are the meteorological data presented in this data article.

**Specifications Table**

**Table:** 

Subject	Environmental Science
Specific subject area	Upper air Meteorological Data for Climatology and Weather prediction
Type of data	Table Image Figure
How the data were acquired	Data were acquired using an assembly of meteorological sensors (temperature sensor, pressure sensor, relative humidity sensor) deployed using weather balloons. Weather packets are then transmitted to a Blockchain database via an outdoor gateway
Data format	Raw Filtered
Description of data collection	A total of 2069 meteorological observations for temperature, pressure, and dew point, with corresponding time, and elevation of capture, were recorded, using Radiosonde equipped with a payload that measures upper air temperature, pressure, and the dew point. The meteorological data logged spans between January 2021 and July 2021
Data source location	City: Uyo Country: Nigeria Latitude and longitude (and GPS coordinates) for collected data: Latitude 4° 52′ 32.477″ N to 5° 5′ 2.288″ N and longitude 7° 47′ 25.785″ E to 8° 0′ 54.393″ E
Data accessibility	Repository name: Mendeley Data Repository Data identification number: 10.17632/xssdssg7z4.1 Direct URL to data: https://data.mendeley.com/datasets/xssdssg7z4

**Value of the Data**

• This dataset provides Spatio-temporal upper air meteorological data of the Uyo local government area of Akwa Ibom State.
• The meteorological data can be compared to data gathered at the same time and date from nearby official meteorological offices, including the Douala airport station in Cameroon.
• Descriptive and diagnostic analysis can be implemented to gain insights into patterns and trends, which can be useful for effective planning and decision-making.
• Predictive and prescriptive analytics can be implemented by employing the dataset to build different machine learning and deep learning algorithms, therefore developing early warning systems for severe weather conditions, such as floods and droughts.
• The dataset can be used in environmental risk assessment and climate change analysis, thus aiding decision-makers to manage, plan and act accordingly.
• Educators can use the dataset for machine learning and deep learning educational activities.

## 1 Objective

Early warning systems are needed to mitigate the effect of climate change in Nigeria and the world at large. There are a few sophisticated prediction systems available, such as the NASA POWER (National Aeronautics and Space Administration's Prediction of Worldwide Energy Resources) [1]. Oloyede et al., [2] presented a descriptive and diagnostic analysis of the NASA POWER temperature data to assess the accuracy of the data in Uyo, which showed a good correlation with ground truth measurement, but the statistical value differs. Furthermore, Scientists from the NAMMA (NASA African Monsoon Multidisciplinary Analyses) data campaign recommend that upper air meteorological data be gathered independently [3]. The main objective of this article is to build on these past insights and make available ground truth measurements of meteorological parameters in Uyo.

## 2 Data Description

**Fig. 1:**
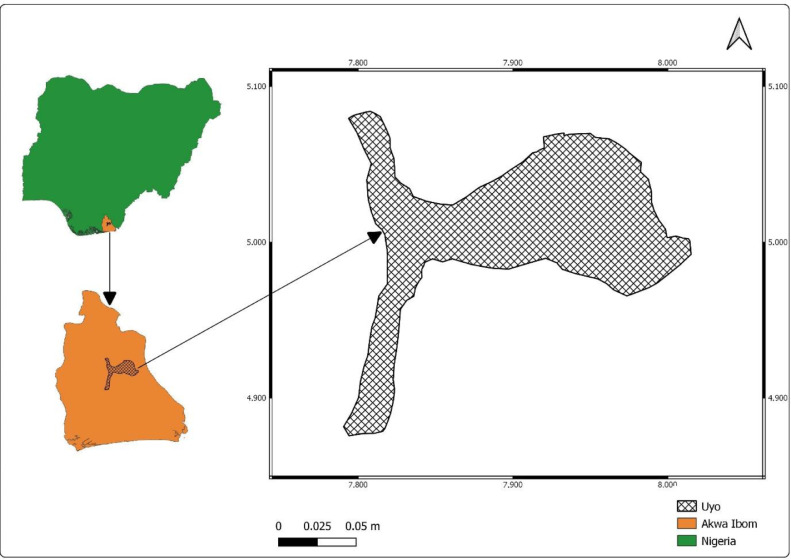
Map of Nigeria showing Akwa Ibom state and Uyo local government area.

Upper air meteorological data for Uyo Local Government Area of Akwa Ibom State is captured and archived using IoT (Internet of Things) and Blockchain technology. Fig. 1 shows the map of Akwa Ibom State in Nigeria, highlighting Uyo Local Government Area, where the radiosonde was launched, and its coverage area.

The dataset [4] consists of 2069 data points and spans through UNIX (UNiplexed Information Computing System) timestamp 1610990623 (January 18, 2021) and 1627293479 (July 26, 2021). Upper air meteorological data plays a vital role in weather prediction and the long term, climate prediction. These data are useful in building algorithms that are capable of forecasting severe flooding and drought, and help to improve the forecasting of Atlantic hurricanes in the seven to fourteen-day timeframe [5]. A few studies have shown that short-term precipitation and vorticity forecasts can be particularly implemented with this type of dataset [6].

The dataset consists of a launch identity, uniquely identifying the payload system responsible for capturing a number of data points. Accordingly, the date/time stamp is captured in Unix timestamp format and corresponding pressure, temperature, dew point, and elevation are captured. Table 1 reports the features contained in the dataset, their description, and their corresponding SI units (International System of Units).

**Table 1:** Features contained in the dataset.

S/N	Feature	Feature Description	SI Unit
1.	Launch_id	This is a unique identification assigned to the payload system responsible for capturing a number of data points in the data set	n/a
2.	Unix_time_s	This feature describes, in Unix timestamp format, the date and time, a parameter is captured	second
3.	Pressure_hpa	This feature describes the pressure measured per time	Hectopascal
4.	Temperature_c	This feature describes the temperature measured per time	Celsius
5.	DewPoint_c	This feature describes the dew point measured per time	Celsius
6.	Elevation2_m	This feature describes the elevation at which the parameters were measured, per time	meter

**Fig. 2:**
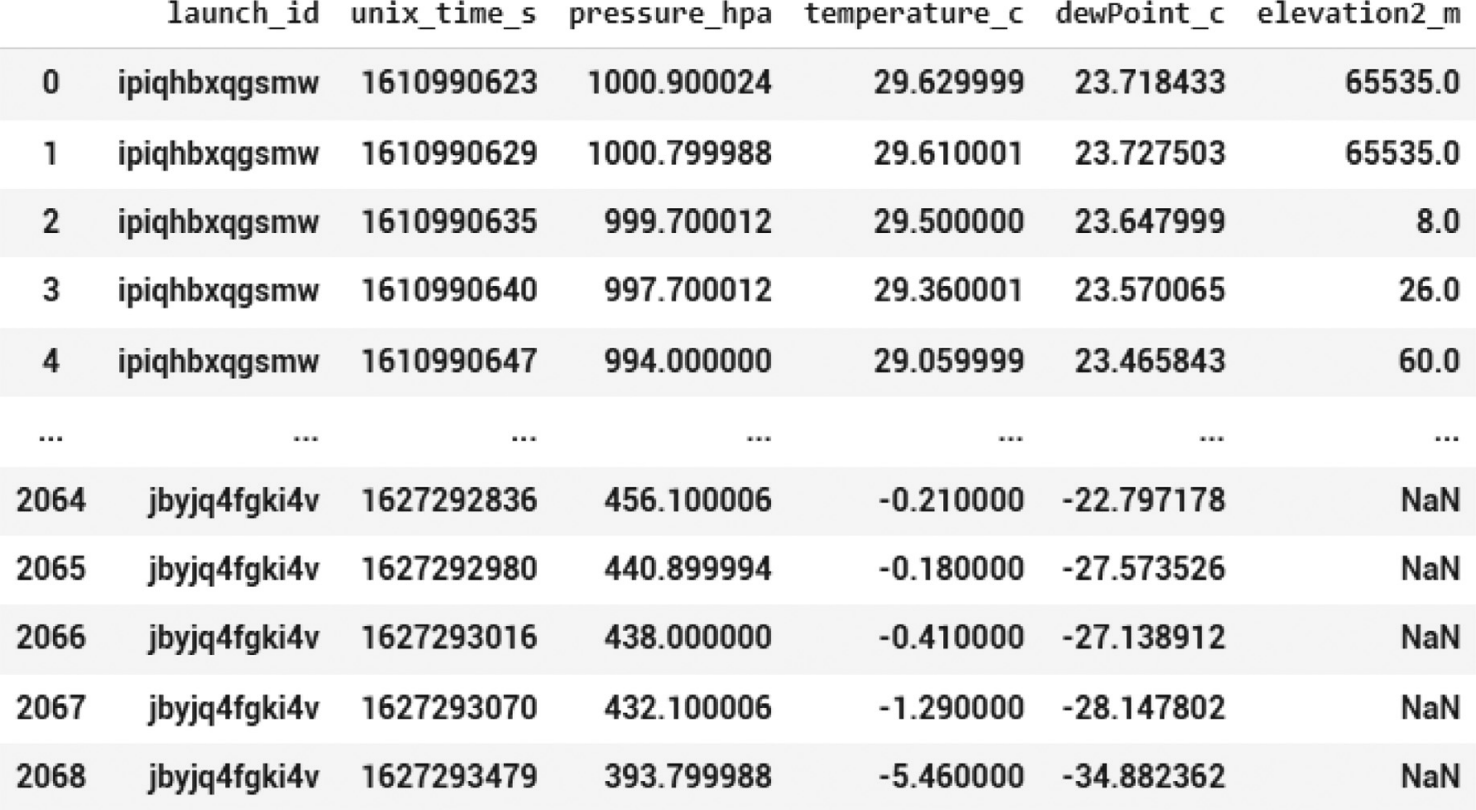
First five and last five rows contained in the dataset.

Fig. 2. Presents a screenshot of the first five and last five rows of the dataset. In this screenshot, the dataset have been filtered, to exclude the columns with entirely null values.

## 3 Experimental Design, Materials and Methods

LoRaWAN (Long-Range Wide-Area Network), a low-power communication protocol designed to connect battery-operated devices to the Internet, is employed for this work. The payload is attached to a weather balloon and deployed so that it keeps floating to a point it becomes more like a satellite with vast footprints capable of establishing communication with tens to hundreds of gateways on the ground. This compensates for times when the balloon drifts away from the launch location due to wind. The LoRaWAN performs best in line-of-sight situations and the SF10 LoRaWAN [7] can do a 15 km range, which is equal to the height at which 30 gm balloons tend to burst, due to low atmospheric pressure.

**Fig. 3:**
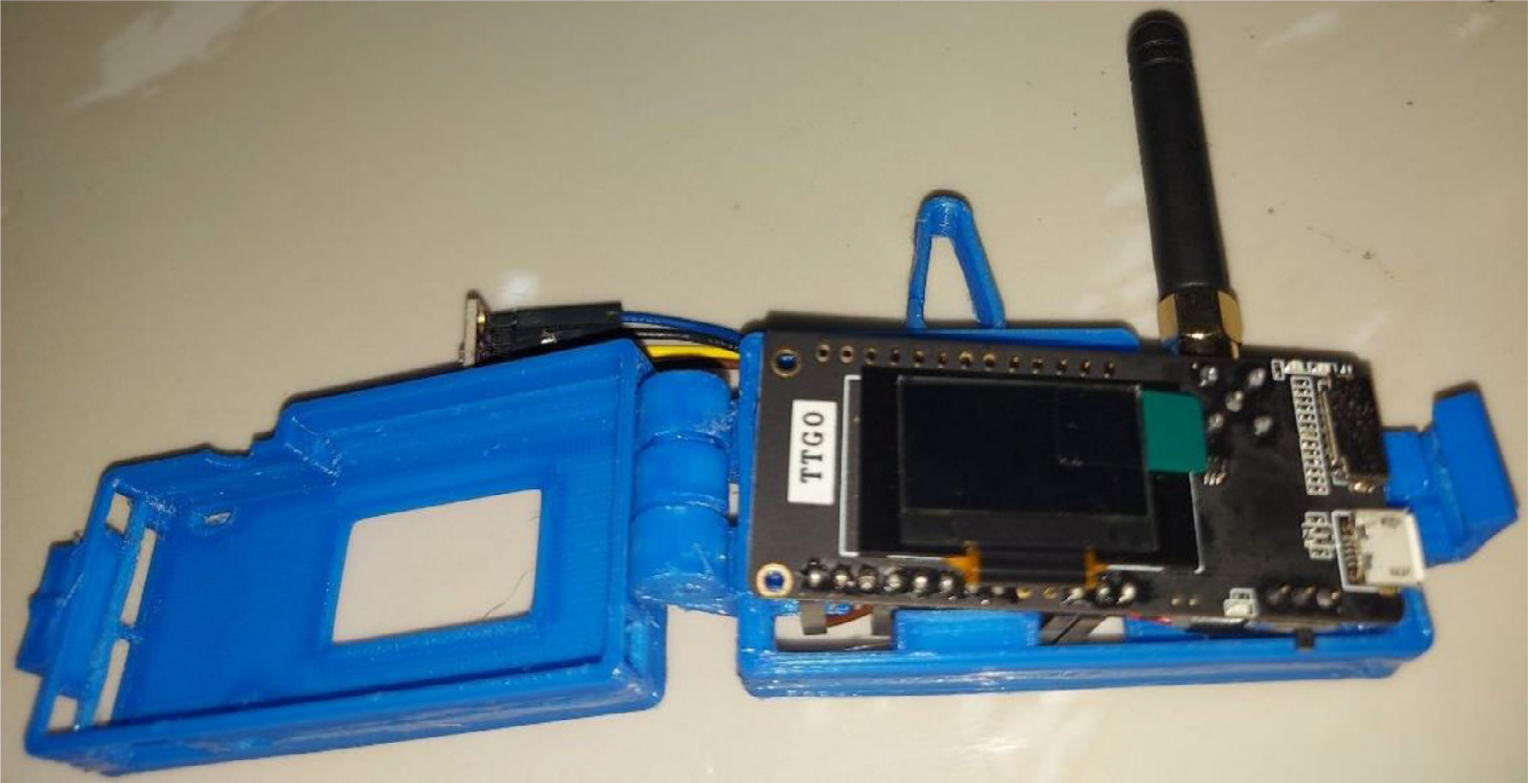
3-D printed enclosure housing the payload.

A 3-D (three-dimensional) printed enclosure is utilized to house an ESP32 TTGO microcontroller with LoRa 868 MHz radio module, a 5 V Bosch BME280 [8] weather sensor that captures ambient temperature, barometric pressure, and relative humidity/dew point, and an Adafruit 350 mah 3.7 V LiPo battery [9] that powers the system. Fig. 3 shows a picture of the payload.

Cloud computing frameworks such as Microsoft's Azure were employed to cater to the software and Blockchain handling. The firmware was put together from a combination of several open-source Arduino libraries. The following is a breakdown of the firmware's components.

(1) Data Transfer: LMIC (LoRaWAN-MAC-in-C) library was utilized to transfer the weather packets over the Helium Blockchain network.
(2) Custom Webpage: WiFi.h library was employed to take care of logins and authentication at the launch. The locally hosted webpage is accessed via http://192.168.4.1 from a web browser or via a QR (Quick Response) code at startup.
(3) Elevation Derivation: As the weather balloon ascends, elevation is determined in real-time by integrating the hypsometric equation over discrete pressure level intervals. It is pertinent to note that the gravitational constant was calculated as a function of latitude because gravity is felt at approximately 1.0% less in West Africa, due to the proximity to the equator.

Furthermore, a Blockchain smart contract is responsible for handling the launch authentication and weather data storage, which are stored as a RAM (Random Access Memory) resource on the Telos Blockchain.

**Fig. 4:**
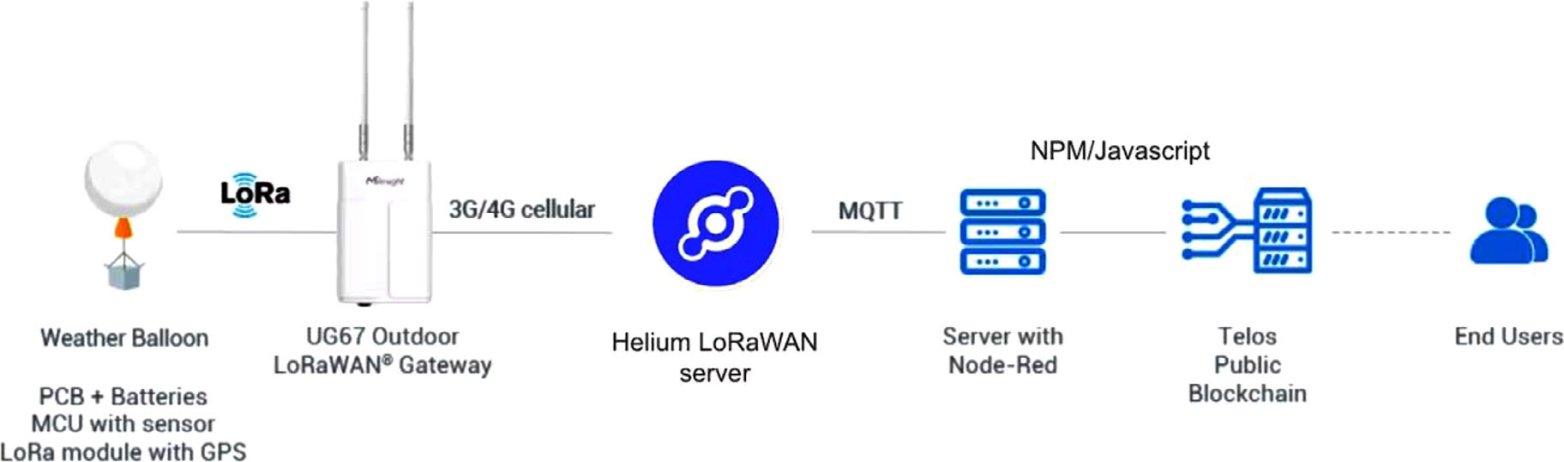
Flow diagram showing the data collection process.

Fig. 4. Shows the entire data-capture and storing process.

**Fig. 5:**
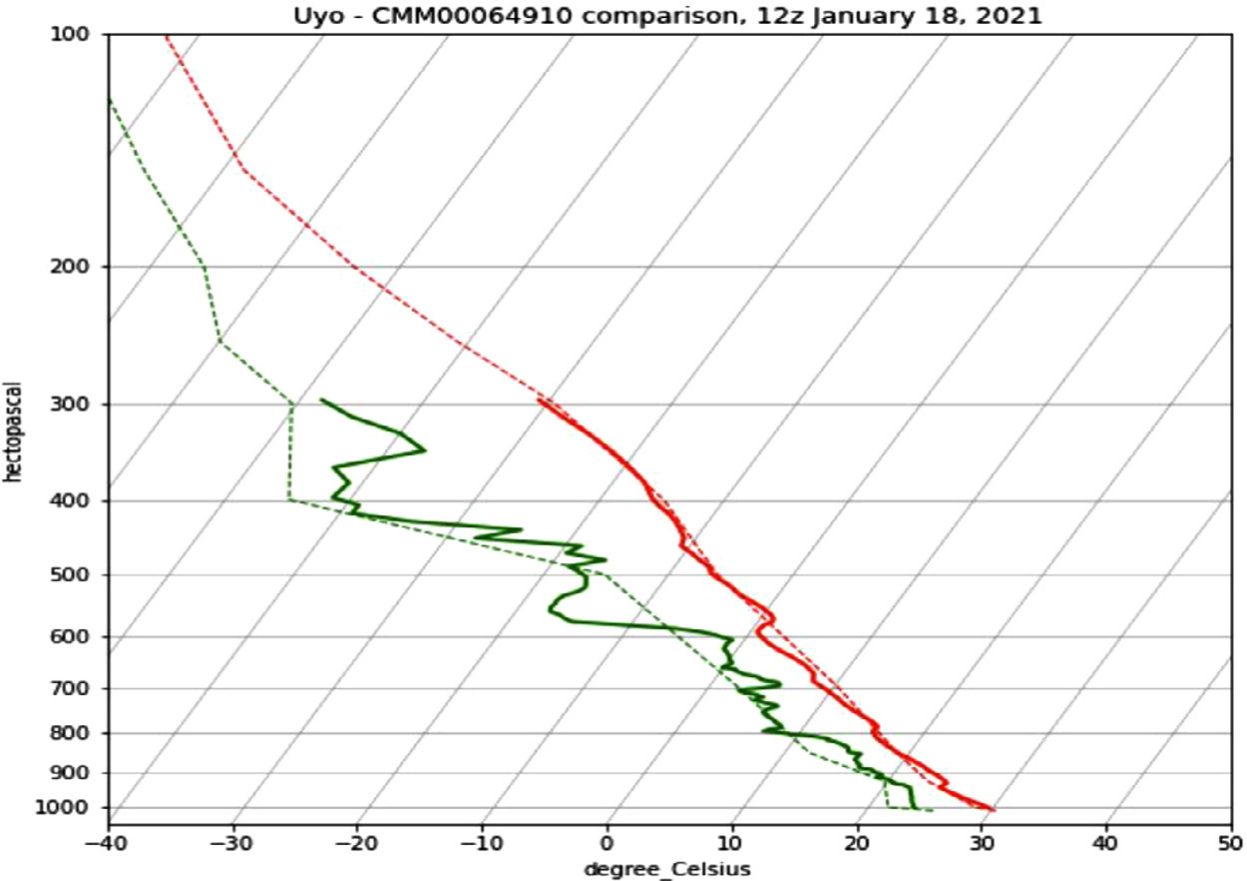
Skew-T plot showing Uyo data (solid line) and the cameroon station CMM00064910 data (dotted line) on january 18, 2021 for temperature (red) and dew point (green).

A few data comparisons to existing meteorological stations in cities like Douala, Accra and Dallas were performed and same presented at the AMS (American Meteorological Society) 2022 annual meeting, in Houston, Texas [10]. Notably, the Cameroon station CMM00064910 located about 200 km Southeast had a few weather balloon releases that coincided at nearly the same time (5 h difference) as a launch from our team in Uyo. The data for the Cameroon station was extracted from the IGRA2 (Integrated Global Radiosonde Archive 2) dataset, and a Skew-T plot of the two radiosonde soundings is presented in Fig. 5. Temperature and dew point are shown in red and green lines respectively, from sea level (1000 hPa) to the troposphere at 6000–10,000 km (≤300 hPa).

The skew-T plot of the two radiosonde soundings shows that the data matched quite well, and even provided higher resolution temperature and dew-point readings during the ascent.

## Ethics Statement

The authors declare that there are no ethical issues with the data presented and that the methodology utilized does not involve animal experiments, human subjects or data collected from social media platforms.

## CRediT authorship contribution statement

**Opeyemi Adamson Oloyede:** Conceptualization, Data curation, Writing – original draft. **Nicolas Lopez:** Methodology, Project administration, Resources. **Simeon Ozuomba:** Supervision, Writing – review & editing. **Philip Asuquo:** Validation, Writing – review & editing. **Emmanuel Essien:** Methodology, Formal analysis. **Alexander Agbu:** Software, Data curation.

## Declaration of Competing Interest

The Authors declare that they have no known competing financial interests or personal relationships that could have appeared to influence the work reported in this paper.

## Data Availability

Upper Air Meteorological Data for Uyo (Original data) (Mendeley Data).
